# Docking of competitive inhibitors to the P2X7 receptor family reveals key differences responsible for changes in response between rat and human

**DOI:** 10.1016/j.bmcl.2015.06.001

**Published:** 2015-08-15

**Authors:** Emily A. Caseley, Stephen P. Muench, Stephen A. Baldwin, Katie Simmons, Colin W. Fishwick, Lin-Hua Jiang

**Affiliations:** a School of Biomedical Sciences, University of Leeds, Leeds, UK; b School of Chemistry, University of Leeds, Leeds, UK

**Keywords:** P2X7 receptor, Adenosine triphosphate, AZ11645373, KN62, SB203580

## Abstract

The P2X7 receptor is a calcium permeable cationic channel activated by extracellular ATP, playing a role in chronic pain, osteoporosis and arthritis. A number of potential lead compounds are inactive against the rat isoform, despite good activity against the human homologue, making animal model studies problematic. Here we have produced P2X7 models and docked three structurally distinct inhibitors using in silico approaches and show they have a similar mode of binding in which Phe95 plays a key role by forming pi-stacking interactions. Importantly this residue is replaced by Leu in the rat P2X7 receptor resulting in a significantly reduced binding affinity. This work provides new insights into binding of P2X7 inhibitors and shows the structural difference in human and rat P2X7 receptors which results in a difference in affinity. Such information is useful both for the rational design of inhibitors based on these scaffolds and also the way in which these compounds are tested in animal models.

## Graphical abstract

**Figure:**
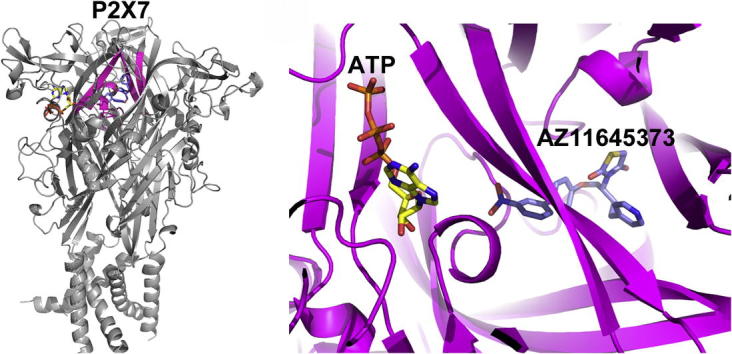

P2X receptors are a family of widely expressed ligand-gated, calcium-permeable cationic channels which are solely activated by extracellular adenosine triphosphate (ATP) in the body.^1^ The seventh member of this family of receptors, P2X7, is expressed primarily in cells of hemopoietic lineage such as macrophages,^2^ monocytes,^3^ lymphocytes,^4^ microglia^5^ and bone cells, and plays a role in processes including inflammation,^6^ bone homeostasis^7^ and immunity.^8^ Whilst the normal activation of this channel is necessary for these processes to occur, P2X7 has also been implicated in playing a role in a wide range of diseases.^9^ The altered expression and/or function of human P2X7 receptors has been related to pathologies including chronic pain conditions,^10,11^ osteoporosis and bone fracture,^12,13^ rheumatoid arthritis,^14^ affective mood disorders^15–17^ and cancers^18^ including chronic lymphocytic leukaemia.^19^ Because of the connection to these debilitating diseases and the appeal of ion channels as pharmaceutical targets, these receptors have come under scrutiny as having potential for drug development. In vivo studies of P2X7 inhibitors in rodents have shown great promise, and pharmacological inhibition of this receptor has been demonstrated to attenuate neuropathic and inflammatory pain,^20^ reduce inflammation in models of rheumatoid arthritis^21^ and to be neuroprotective and to significantly reduce the number of amyloid plaques in the brain in mice models of Alzheimer’s disease.^22,23^ As a result several P2X7 receptor-specific compounds have been developed by pharmaceutical companies to treat chronic conditions such as rheumatoid arthritis, osteoarthritis and chronic obstructive pulmonary disease.^24^ However, despite much time and effort being invested in the development of these compounds, the results in phase 1 and 2 clinical trials^25–28^ have been disappointing. A deeper understanding of the mode of binding of these inhibitors will aid in the development of these potential drugs. An important consideration for the development of these inhibitors must also be the structural differences seen between rodent and human P2X7 receptors. This would create problems when testing in an animal model situation unless appropriate measures are made, for example, the generation of a transgenic rat model. A thorough understanding of the P2X7 receptor structure–function relationship is therefore crucial for the development of P2X7 receptor ligands as therapeutics as well as advancing our understanding of this receptor.

A major breakthrough in the understanding of the structure of the P2X receptors arrived when the crystal structure of the zebrafish P2X4 receptor was solved.^29^ This structure gave an unprecedented insight into the structure of this receptor in the closed state, which was followed by its structure in the ATP-bound open state.^30^ This structure has guided homology modelling which can be used as a basis for further in-depth structural studies of the P2X7 receptor.

A number of P2X7-specific antagonists have been identified, several of which display desirable drug-like properties. Many of these display high levels of affinity for the P2X7 receptor, acting at concentrations in the low nanomolar range. These compounds have the potential to act as a springboard for even more effective antagonists. However, the opportunities for development are somewhat limited. This is due in part to the lack of detailed knowledge concerning the binding site of these antagonists. Whilst several existing compounds are known to act as competitive inhibitors, binding at the ATP binding pocket about which we know a large amount, there are a number of non-competitive P2X7-specific inhibitors. Where these compounds bind is less well understood, and determining the site at which these compounds interact with the receptor requires further investigation.

To this end we investigated the mode of binding of this family of P2X7 antagonist inhibitors based on in silico approaches. Structural models of P2X receptors were produced based on the zebrafish P2X4 crystal structure in the closed state (Protein Data Bank code 4DW0) using Modeller version 9.12^31,32^ (Fig. 1A). Docking studies were carried out using AutoDock version 4.2.^33^ All necessary files were prepared using AutoDock tools. The target cavity file for each docking simulation consisted of a 15 Å sphere in the extracellular domain of the P2X7 receptor, centred around the amino acid at position 95, since this had previously been reported to be involved in binding.^34,35^ Ligands were docked using standard parameters and a standard Lamarckian genetic algorithm. Dockings were visualised using AutoDock tools and were clustered using 2 Å RMS. The most populated/lowest energy poses were examined.

Three P2X7-specific inhibitors (Table 1) were docked into the homology model of the human and rat P2X7 receptors (based on the zebrafish P2X4 receptor^29^) using AutoDock in order to investigate potential binding sites of these compounds. 100 different docking conformations were obtained for each compound with the ten lowest predicted binding energy scores being selected for further analysis.

Three commercially available and widely used P2X7-specific antagonists were docked as part of this study; SB203580, KN62 and AZ11645373 (Table 1). The results of these molecular docking simulations suggested a similar binding mode for each of the P2X7 antagonists. Figure 1 shows the general area at which all three antagonists were predicted to act upon the receptor, which is proximal to the ATP binding site. This pocket is located at the interface between the neighbouring subunits, in close proximity to the α2 helix and two of the β-strands (β13 and β14) of the upper body domain (Fig. 1B). This pocket is found in the inner vestibule of the receptor and for the largest of the inhibitors, KN62, amino acids from two different chains appear to interact with the inhibitor. Residues within this region play a key role in determining the orientation of the antagonists. Such residues which come into immediate contact with the docked antagonists include the hydrophobic Phe95, Tyr295, Tyr299 and Tyr300 in addition to the positively charged Lys297, which likely play an important role in determining the orientation of bound antagonists such as those docked in this study.

Of these important residues in the binding site, Phe95 appears to have a key role in determining the orientation of each compound. This residue is predicted to form pi-stacking interactions with aromatic rings within the structures of the three antagonists (Fig. 2A, C and E). Interestingly, previous studies have identified Phe95 as determining the species-specific activity of some P2X7-specific antagonists.^34,35^ This previous work investigated the mechanism of action of species-specific P2X7 inhibitors. The first of these studies found that mutating Phe95 in the human receptor to the Leu found in the rat greatly reduced the inhibitory activity of KN62 and SB203580,^34^ whereas the second found that this was also the case with AZ11645373.^35^ The three antagonists docked in this study all display species-specific activity to varying degrees. SB203580 acts as a low-affinity, non-competitive inhibitor of the human receptor, with an IC_50_ of approximately 5 μM, but does not act at the rat receptor.^36^ KN62 inhibits the human receptor with an IC_50_ of ∼100 nM but similarly does not act on the rat^37^ receptor. AZ11645373 inhibits human P2X7 with an IC_50_ of ∼90 nM whereas this compound is over 500-fold less effective at inhibiting the rat receptor and results in less than 50% inhibition at concentrations of 10 μM.^38^ In the rat P2X7 receptor the equivalent residue to Phe95 is a leucine, which due to its lack of aromatic side chain will lose the stacking interaction of these compounds with the receptor. As such we carried out docking with these molecules in the rat receptor to accompany the experiments in the human P2X7 receptor. The results were strikingly different to those seen in the human counterpart; with significantly lower predicted binding affinities (in the millimolar range for the rat receptor compared to nano- or micromolar range for human) as well as notably different predicted binding positions within this site although agonist efficacy may also play a role.

As shown in Figure 2B, D and F, the pi-stacking interactions seen between Phe95 and the aromatic rings of the inhibitors are lost in the same site in the rat P2X7 receptor. This provides striking evidence for the essential role of this residue in determining the species-specific activity of these compounds. Sequence alignment of residues within 8 Å of the predicted binding site shows that Phe95 is the only residue which is not conserved between the human and rat receptor located in a region which may interact with the inhibitors (Fig. 3). The importance of this residue is further validated by previous studies which used a human–rat chimeric receptor whereby a leucine replaced Phe at this position in the human P2X7 receptor, greatly reducing the efficacy of these three inhibitors.^34,35^ Conversely, introducing the L95F mutation into the rat receptor has been shown to have a varied impact on the ability of the compounds described above to inhibit the rat P2X7 receptor.^34^ SB203580 changes from having no effect at the wild-type receptor to acting as an antagonist of the L95F rat receptor, albeit to a lesser degree than the human receptor. Additionally, the L95F mutation allows KN62 to modestly inhibit responses when lower concentrations of ATP are applied.

The inhibitor binding site identified in these docking simulations and importantly the mode of binding, builds on the previous knowledge we have concerning the location at which these compounds act, which is currently limited. This site is proximal to the ATP binding site, buried within the receptor (Fig. 1). It has previously been shown that these antagonists are non-competitive inhibitors and do not directly obstruct the ATP binding site.^35,36,39^ However, these compounds bind relatively closely to the ATP binding pocket. Studies combining molecular modelling simulations of P2X receptors and experimental evidence have shown that flexibility in this region is necessary for the activation of these receptors following agonist binding.^40,41^ Inhibition of conformational flexibility of the ATP binding pocket has been shown to result in depressed responses to P2X receptor agonists,^40^ in addition to further evidence which indicates that the tightening of the ATP-binding jaw induces opening of the P2X channel.^41^ It has previously been suggested that competitive P2X receptor antagonists prevent receptor activation by means of preventing this movement of binding pocket tightening.^41^ As such it seems reasonable to suggest that the binding of the antagonists examined in this study to a site behind the ATP-binding pocket may inhibit channel activation by preventing this vital conformational flexibility of the P2X receptor.

The simulations carried out in this study highlight a potential binding conformation for a series of P2X7-specific compounds and provide new insights into existing studies. This is of great interest in terms of drug development as the human P2X7 receptor has been identified as a desirable target for therapeutic compounds to treat the above-mentioned disease conditions. A more in-depth understanding of the binding site of existing antagonists opens the door for further rational development of these already effective compounds as P2X7 receptor inhibitors.

## Figures and Tables

**Figure 1:**
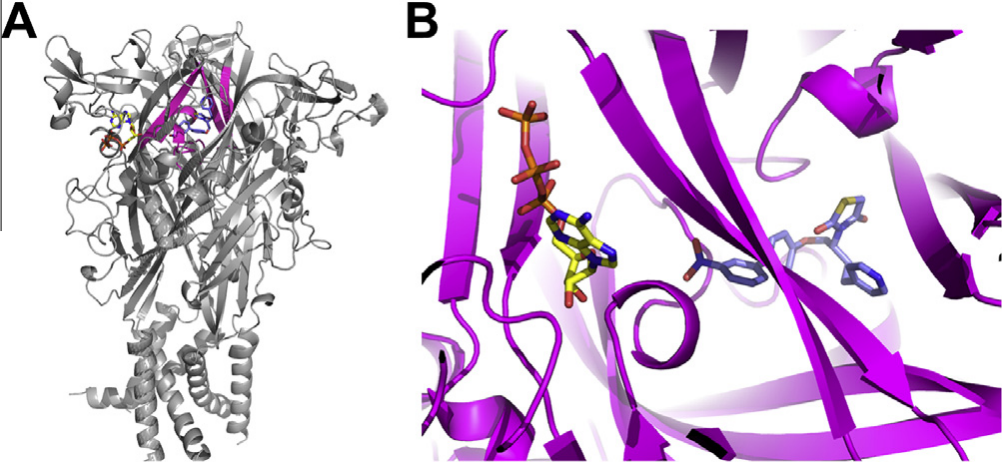
Predicted antagonist binding site in relation to the known ATP binding site. (A) Docking of SB203580 and ATP into the human P2X7 receptor, with residues proximal to the antagonist binding site shown in purple. (B) Enlarged view of the antagonist binding region highlighted in purple.

**Figure 2:**
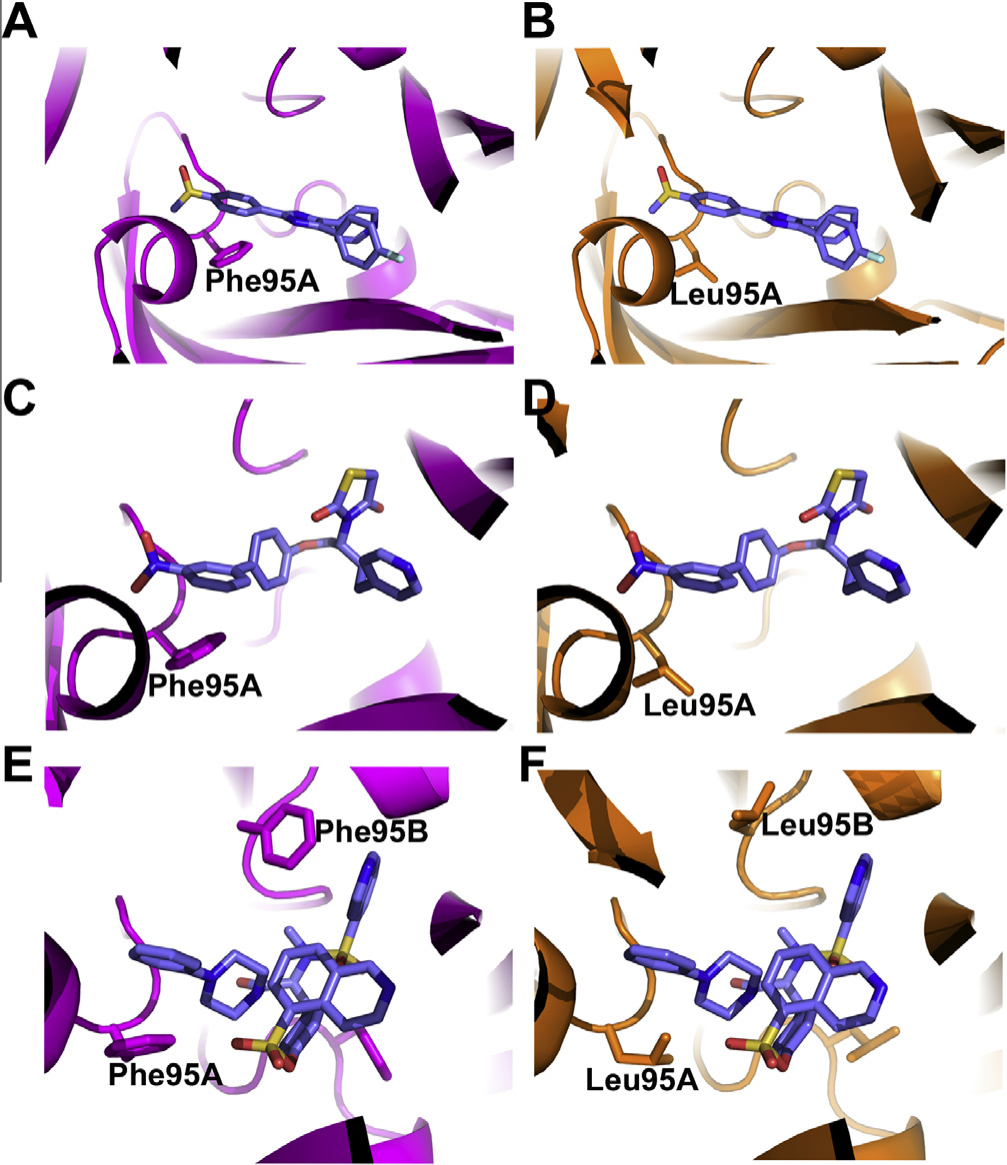
Docking of SB203580, AZ11645373 and KN62 into the human (A, C and E) and rat (B, D and F) P2X7 receptors, respectively. Residues Phe95 and Leu95 as well as docked inhibitors are depicted in stick format. Residues labelled as A and B are from different subunits of the same receptor.

**Figure 3:**
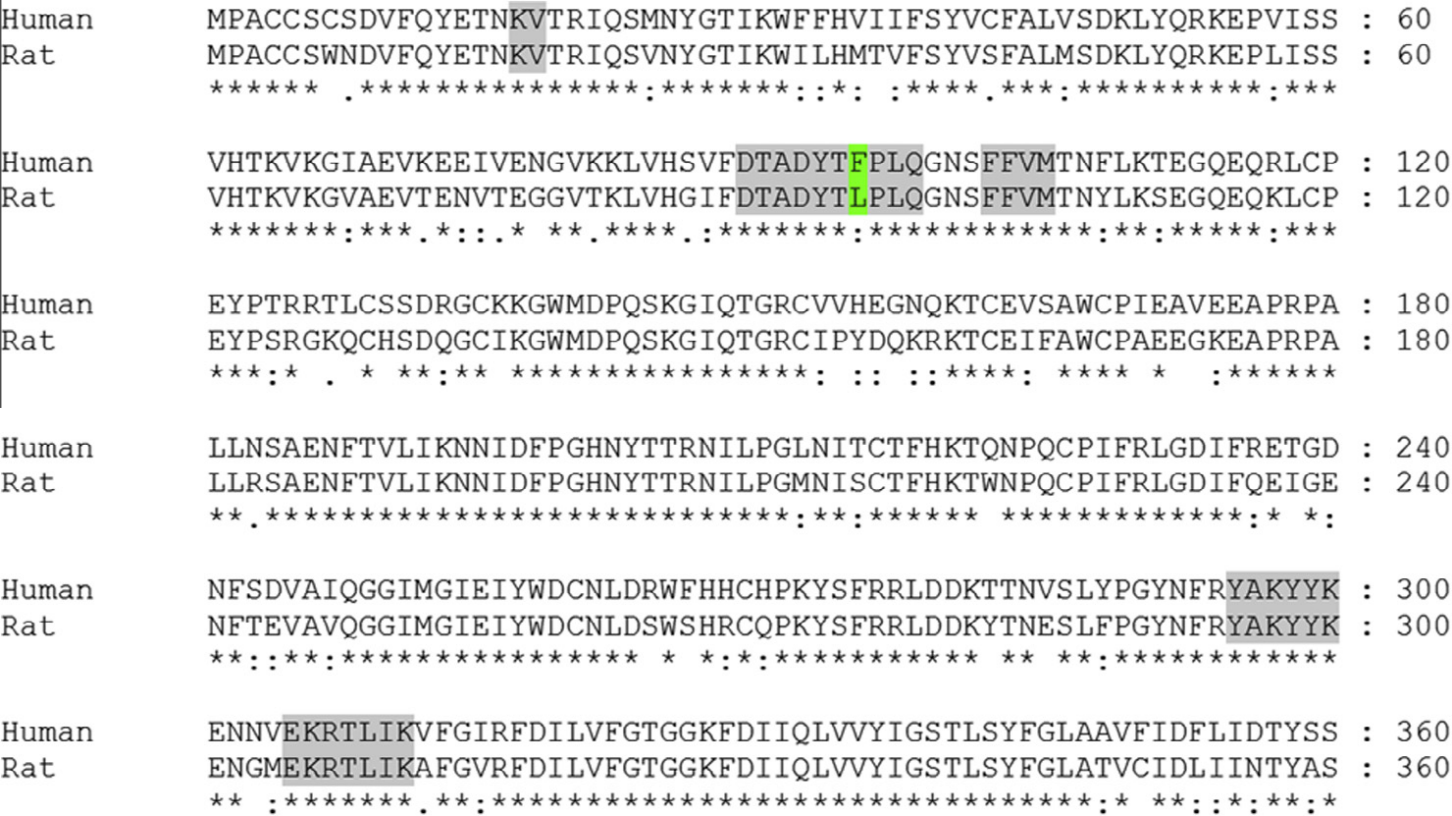
Sequence alignment of the first 360 amino acids of the rat and human P2X7 receptors with residues which are within 8 Å of the predicted ligand binding site highlighted in grey. Residues which differ between the two sequences are highlighted in green.

**Table 1:** Activity data for human P2X7 receptor inhibitors

Compound ID	Structure	IC_50_ at human	IC_50_ at rat	Activity in further species	References
SB203580		∼5 μM	>30 μM	None reported	
KN62		100 nM	>100 μM	Rhesus macaque monkey, IC_50_ 86 nM Guinea pig, IC_50_ 130 nM Dog, IC_50_ 10 nM	42,43
AZ11645373		31 nM	>100 μM	Rhesus macaque monkey, IC_50_ 23 nM	42
